# Self-rated health and multimorbidity in patients with type 2 diabetes

**DOI:** 10.1177/13591053211001419

**Published:** 2021-03-25

**Authors:** Kanayo Umeh

**Affiliations:** Liverpool John Moores University, UK

**Keywords:** Beliefs, diabetes, mediator, obesity, perception

## Abstract

The relationship between multimorbidity and self-rated health is well established. This study examined self-rated health in relation to multimorbidity, glycaemia and body weight specifically in adults with type 2 diabetes. Bootstrapped hierarchical logistic regression and structural equation modelling (SEM) were used to analyse survey data from 280 adults with type 2 diabetes. The odds of ‘fair/bad/very bad’ self-rated health increased 10-fold in patients with three (OR = 10.11 (3.36–30.40)) and four conditions (OR = 10.58 (2.9–38.25)), irrespective of glycaemic control (*p* < 0.001). The relationship between multimorbidity and perceived health was more pronounced in male patients. SEM generated a model with good fit, χ^2^ (CMIN) = 5.10, df = 3, *p* = 0.164, χ^2^ (CMIN)/df = 1.70, RMSEA = 0.05, CFI = 0.97, TLI = 0.95 and NFI = 0.94; self-rated health mediated relations between multimorbidity and BMI. Overall, this study highlights the potential of self-rated health to mediate relationships between multimorbidity and BMI, but not glycaemic control, in adults with type 2 diabetes.

## Background

Multimorbidity (defined as having ⩾2 chronic conditions) (Chiang et al., 2018) requires the management of disease clusters, which can complicate patient care (Mair and May, 2014). Multimorbid patients need to juggle different medication regimens (e.g. multiple dosing schedules), which may reduce adherence (Harris et al., 2014), and increase the risk of premature mortality (Chiang et al., 2020). Multimorbidity is present in the majority (up to 90%) of people diagnosed with type 2 diabetes (Huntley et al., 2012; Teljeur et al., 2013). Thus, there is growing interest in disease clusters in type 2 diabetes, and the implications for patient outcomes, including glycaemic control (Chiang et al., 2018, 2020). The association between multimorbidity and glycaemia (based on the haemoglobin A1c (or HbA1c) diagnostic test) is unclear (Chiang et al., 2018). Furthermore obesity, a major risk factor for type 2 diabetes (e.g. insulin resistance) (Agha and Agha, 2017), more than doubles the odds of multimorbidities (Agborsangaya et al., 2013). Given the heightened interest in how multimorbidity relates to glycaemia (Chiang et al., 2018) and body weight (Madlock-Brown and Reynolds, 2019), there is a need to identify mechanisms underpinning these associations.

### Self-rated health

The relationship between self-rated health and multimorbidity is well documented (Bustos-Vazquez et al., 2017; Galenkamp et al., 2011; Ishizaki et al., 2019; Kaneva et al., 2018; Mavaddat et al., 2014; Song et al., 2018). For example, a population-based study of 25,268 middle-aged and older adults (aged 39–79 years) recruited from general practice registers (Mavaddat et al., 2014) found the odds of ‘moderate/poor’ self-rated health was approximately twice as high in people with two or more conditions, compared to those reporting only one condition. Research has also found a link between self-rated health and glycaemic control. For example, analysis of data from 606 patients with type 2 diabetes (median age 65.6 years) found that poorer perceived health was associated with higher HbA1c despite adjusting for covariates such as symptoms, antidiabetic medication and fatigue (Nielsen et al., 2011). There is also a robust (albeit conditional) relationship between self-rated health and body weight. An analysis of data from 70 countries (160,099 participants) found that body mass index (BMI) was negatively associated with poor self-rated health, in both men and women, from low-income countries (the relationship was reversed in women from middle-income countries) (Wang and Arah, 2015). Thus, the association between perceived health and bodyweight depended on gender and socio-economic background. Regardless, most of the aforementioned studies on self-rated health and multimorbidity used national survey data, or samples from the general population. Few investigations have focused on patients with a specific chronic disease. It remains unclear how self-rated health relates to multimorbidity, and concomitant glycaemia and obesity, in patients with type 2 diabetes.

### Glycaemia

Glycaemic control in people with diabetes is typically assessed using the HbA1c test (gauges average glucose levels in the past 2–3 months) (Sherwani et al., 2016). Although multimorbidity correlates with glycaemic control, evidence is mixed. A recent systematic review found that 10 of 14 studies reported no significant relationship between multimorbidity and HbA1c; by contrast, 4 out of the 14 studies found that higher levels of multimorbidity were associated with elevated HbA1c (Chiang et al., 2018). The discrepancies may be partly attributable to variance in self-rated health, since perceived health is related to both multimorbidity (Mavaddat et al., 2014) and glycaemia (Abualula et al., 2018; Nielsen et al., 2011). For example, it is possible people living with more chronic conditions develop poor perceptions of health that in turn inhibit diabetes self-management practices (Idler and Benyamini, 1997), resulting in poor glycaemic control (Nielsen et al., 2011). Thus, there is a need to understand, not just how multimorbidity relates to self-rated health (Mavaddat et al., 2014), but also how the latter influences multimorbidity–HbA1c relations. Hitherto, this issue has not been addressed in the literature (Chiang et al., 2018).

### Obesity

Several population-based studies have demonstrated a robust relationship between multimorbidity and body weight (Agrawal and Agrawal, 2016; Booth et al., 2014; Kivimaki et al., 2017). For example, an analysis of cross-sectional data from 40,166 participants across six countries found that prevalence of non-communicable disease multimorbidity was 1.5 times higher in people with obesity, compared to people of normal weight (Agrawal and Agrawal, 2016). A study of the electronic health records of 300,006 adults aged ⩾30 years found a positive association between multimorbidity and obesity (Booth et al., 2014). Another investigation analysed pooled data on body weight and cardio-metabolic multimorbidity from 120,813 adults, across 16 cohort studies (Kivimaki et al., 2017). The probability of multimorbidity increased given elevated BMI scores; the risk of multimorbidity was double in people who are overweight, and over 10 times in those with severe obesity, compared to those of normal weight. While self-rated health has been strongly associated with body weight (Wang and Arah, 2015), it is unclear to what extent the former explains the multimorbidity–BMI relationship. As suggested earlier, people experiencing multiple illnesses may evaluate their health negatively, thereby negating self-care practices essential for weight control. Alternatively, obesity may elicit poor perceived health, leading to health-compromising behaviours that accentuate both multimorbidity and obesity (Idler and Benyamini, 1997). Further research is needed to test these potential mediator effects.

### Research objectives

The aims of this investigation were to (a) assess the association between multimorbidity and self-rated health in patients with type 2 diabetes and (b) analyse the structural relationships between multimorbidity, HbA1c and body weight, whereby self-rated health is treated as a mediating factor. The study addressed two specific research questions:

1. Is multimorbidity associated with self-rated health in people diagnosed with type 2 diabetes? Based on previous research with a general population of patients (Mavaddat et al., 2014), it was hypothesised that the odds of poor perceived health is significantly higher in patients with multimorbidity (i.e. ⩾2 conditions), compared to patients without multimorbidity (Hypothesis 1). It was expected that this association persists even after accounting for glycaemia (HbA1c), body weight (BMI) and other clinical characteristics.
2. Is the structural relationship between multimorbidity and both HbA1c and BMI in people with type 2 diabetes mediated by self-rated health? Since perceived health has been associated with multimorbidity (Bustos-Vazquez et al., 2017), glycaemia (Nielsen et al., 2011) and BMI (Wang and Arah, 2015), it was hypothesised that multimorbidity is indirectly related to both HbA1c and body weight, mediated by self-rated health; greater multimorbidity, HbA1c and BMI levels are underpinned by poor perceived health (Hypothesis 2). A direct association between multimorbidity and HbA1c was not expected due to weak support from multiple studies (Chiang et al., 2018).

## Methodology

### Ethics

Ethics approval for this study was provided by the university research ethics committee, based on a wider project involving the Health Survey for England (approval number 16/NSP/035). The study was performed in accordance with ethical standards as laid down in the 1964 Declaration of Helsinki and its later amendments or comparable ethical standards.

### Study sample and design

**Figure 1.**
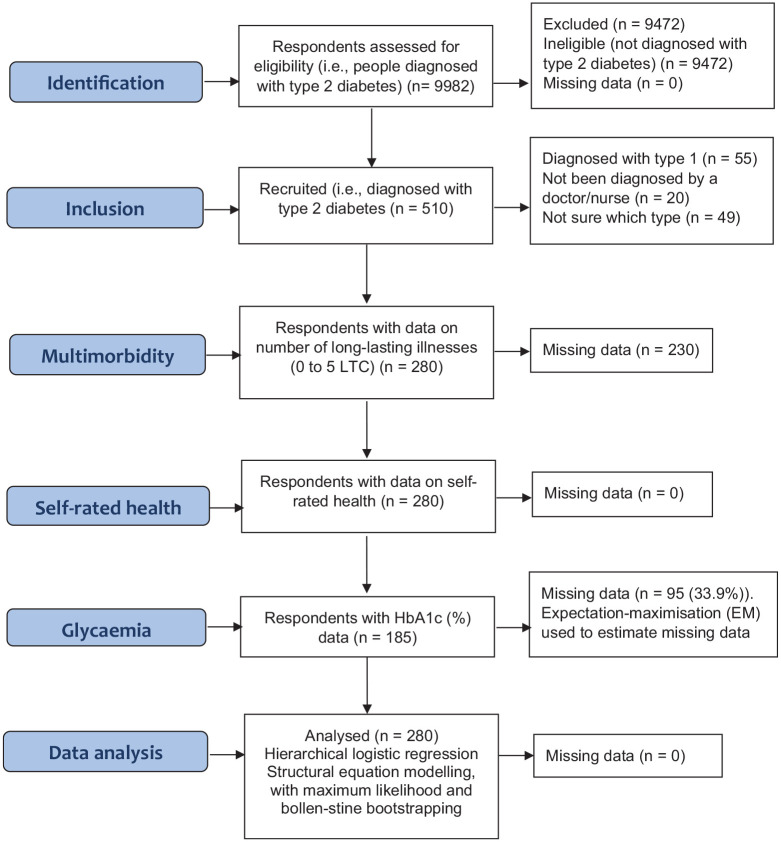
STROBE flow diagram.

Patient data was extracted from the 2017 *Health Survey for England*, a national population review conducted annually in the UK (Mindell et al., 2012). Inclusion criteria were as follows: (1) had been diagnosed with type 2 diabetes by a doctor or nurse, at the time of the survey, (2) was aged 16 years or older, (3) provided data on number of chronic conditions, and self-rated health. Although HbA1c ⩾ 6.5% is used to diagnose diabetes, this threshold was not an inclusion requirement due to potentially misleading fluctuations in patients with type 2 diabetes (e.g. falsely lowered HbA1c that does not accurately reflect true average glycaemia, and suggesting a participant does not have diabetes) (Radin, 2014). Exclusion criteria were as follows: (1) had been diagnosed with type 1 diabetes by a doctor or nurse, at the time of the survey; (2) aged below 16 years; (3) had no data on number of chronic conditions, and self-rated health. The study recruitment sequence is outlined in Figure 1. Overall, 9472 (94.8%) respondents were ineligible. Of the remainder, 510 (5.1%) had been diagnosed with type 2 diabetes, and of this number 280 (2.8%) patients met the eligibility criteria.

### Self-rated health

Self-rated health has historically been measured using three to five ordinal categories depicting favourable, neutral and unfavourable evaluations (e.g. ‘poor’, ‘fair’, ‘good’) (Idler and Benyamini, 1997). The national survey employed five options: ‘very bad’ (0), ‘bad’ (1), ‘fair’ (2), ‘good’ (3) and ‘very good’ (4). Measures are typically collapsed into a dichotomous variable, with just two categories (e.g. ‘bad/fair’ vs ‘good’) (Bourne, 2009; Manor et al., 2000), due to the paucity of patients in the ‘bad’ (12.9%) and ‘very bad’ (5%) categories (Mavaddat et al., 2014)).

### Socio-demographics and morbidity

Age was assessed using eight bands (e.g. 16–24, 25–34, up to 65–74, ⩾75), dichotomised as ‘younger’ (0–64 years) and ‘older’ (⩾65). Socio-economic class was based on eight groupings (e.g. higher/lower managerial, intermediate, semi-routine/routine, unemployed), categorised as ‘lower’ (coded 0) versus ‘higher’ (coded 1). Multimorbidity was based on the number of reported chronic conditions (up to 5). Five dummy variables were created, with zero (0) multimorbidity treated as the reference category: one condition (1 (coded 1) vs 0 (coded 0)), two conditions (2 (coded 1) vs 0 (coded 0)), three conditions (3 (coded 1) vs 0 (coded 0)), four conditions (4 (coded 1) vs 0 (coded 0)) and five conditions (5 (coded 1) vs 0 (coded 0)). For descriptive purposes, an additional dummy variable was generated; multimorbidity absent (<2 conditions (coded 0)) versus present (⩾2 conditions (coded 1)).

The data were also reviewed for comorbidities. Four relevant chronic conditions were identified: asthma/COPD, heart disease, obesity and anxiety/depression. All are well documented chronic complications of diabetes (Ehrlich et al., 2010; Khan et al., 2019; Smail, 2019). Respondents indicated if they had ever had a heart attack (including myocardial infarction or coronary thrombosis); ‘yes’ (coded 1), ‘no’ (coded 0). They also stated if they had taken any prescribed asthma/COPD medications in the past 7 days; ‘no’ (coded 0) or ‘yes’ (coded 1). Presence of anxiety/depression was measured using the EQ 5D scale, from the EuroQol group (Rabin and de Charro, 2001); ‘no’ (coded 0) or ‘yes’ (coded 1). Obesity was based on BMI figures, and dichotomised as: <30 (normal, overweight (coded 0)) versus ⩾30 (obese (coded 1)).

### Physiological measurements

Blood samples were drawn during the nurse visit, and tested for glycated haemoglobin (HbA1c), and total/HDL cholesterol. HbA1c data was available in both IFCC (International Federation of Clinical Chemistry) units of mmol/mol and also DCCT (Diabetes Control and Complications Trial) percentages. It was decided to analyse DCCT units (%), albeit IFCC calibration is more common in Europe (Goodall, 2005). The HbA1c % data was dichotomised; as non-diabetes (and prediabetes) falls within the 4.0%–6.4% range, 6.5% was used as the threshold (<6.5% (coded 0) vs ⩾6.5% (coded 1)). Since it is recommended that people with diabetes maintain HbA1c levels below 7%, above which the risk of complications increases markedly, an additional dummy variable was created using this threshold (<7.0% (coded 0) vs ⩾7.0% (coded 1)). Other clinical characteristics were also dichotomised, again with unhealthy values coded as 1: HDL or ‘good cholesterol’ (⩾1 mmol/L (coded 0) vs <1 mmol/L (coded 1)); total cholesterol (⩽5 mmol/L (coded 0) vs > 5 mmol/L (coded 1)). Although the survey asked patients whether or not they had been diagnosed with hypertension (high blood pressure), systolic and diastolic blood pressure readings were also available. Thus, these characteristics were coded separately due to their differential impact on health (e.g. systolic pressure is a more important predictor of mortality in older adults) (Taylor et al., 2011); systolic (⩽120 mm Hg (coded 0) vs >120 mm Hg (coded 1)); diastolic (⩽80 mm Hg (coded 0) vs > 80 mm Hg (coded 1)).

### Data analysis

Logistic regression was used to test Hypothesis 1. Power analysis was performed using G*Power 3.1.7 protocols (Faul et al., 2009), in order to determine the minimum required sample size, given an alpha level of 0.05, a power of 0.80 (Gelman and Hill, 2006), a large effect size (odds ratio = 6.87) (Mavaddat et al., 2014) and a one-tailed test, where X parm п was based on the smallest multimorbidity count. These parameters generated a minimum sample size of *N* = 226. A hierarchical procedure was used for variable entry, in order to evaluate the contributions of predictors above and beyond previously entered variables.

It was decided to test four models: Model 1 (self-rated health = Intercept + Age + Gender), Model 2 (self-rated health = Intercept + Age + Gender + multimorbidity), Model 3 (self-rated health = Intercept + Age + Gender + multimorbidity + HbA1c/blood pressure/lipids) and Model 4 (self-rated health = Intercept + Age + Gender + multimorbidity + HbA1c/blood pressure/lipids + comorbidities). Of interest was whether any significant associations between multimorbidity and self-rated health (Model 2) persisted after accounting for physiological risk factors (e.g. Hba1c) (Model 3), and specific comorbidities (Model 4). This hierarchy was based on the theoretical premise that comorbidity is either embedded within the broader concept of multimorbidity (and hence does not explain additional variance in outcome data), or is a completely different entity from multimorbidity (in which case comorbidity may predict additional variance, over and beyond that attributable to multimorbidity) (Lefevre et al., 2014). Nevertheless, this proposition remains the subject of ongoing debate about the definition of multimorbidity, which is beyond the scope of this paper (Lefevre et al., 2014).

SEM was performed to test Hypothesis 2 (i.e. the relationship of multimorbidity with HbA1c and BMI is mediated by self-rated health). A sample size of 200 or larger is recommended for SEM models (Kline, 2011). The main test of model fit (χ^2^ goodness-of-fit) is affected by sample size, but performs adequately given 200–300 participants (Kenny, 2012). The present analysis was based on data from the whole sample (*N* = 280), using IBM AMOS SPSS statistical pack-age (Version 26). The modelling was conducted as path analysis, and rectangles represented measured variables. It was decided to use maximum likelihood estimation, which meant treating both multimorbidity (i.e. number of conditions; 0–5) and self-rated health (0 = ‘bad/very bad’ (0), ‘fair’ (1), ‘good’ (2) and ‘very good’ (3)) as continuous variables. Maximum likelihood estimation assumes multivariate normality. Thus, all key variables were tested for skewness, and kurtosis, based on the general principle that skewness between −0.5 and 0.5 indicates symmetrical data, and kurtosis close to 0 (less than 3.00) denotes a normal distribution. The skewness for HbA1c, BMI, self-rated health and multimorbidity varied from 0.16 to 1.25 indicating some asymmetry. Kurtosis varied from −0.65 to 1.60, which, although <3.00, suggested mildly mesokurtic distributions, with both platykurtic (kurtosis < 0) and leptokurtic (kurtosis > 0) bias (Westfall & Henning, 2013). Consequently, it was decided to perform SEM using the Bollen-Stine bootstrap procedure, with 200 resamples, for testing the null hypothesis that the model is correct. The Bollen-Stine method is a modified bootstrap technique for the χ^2^ goodness of fit statistic, which provides a means to adjust for bias in standard error and fit parameters due to non-normal data.

## Results

### Sample characteristics

Overall, 375 patients had been diagnosed with diabetes, of which 280 (74.67%) were told by a doctor or nurse they had type 2 diabetes (Figure 1). HbA1c data was unavailable for 95 patients (33.9%), otherwise all participants had complete data for self-rated health, BMI, multimorbidity, comorbidities (heart disease, asthma/COPD, anxiety/depression), demographics (age, gender, social class) and physiological covariates (diastolic/systolic blood pressure, total/HDL cholesterol). The final study sample thus comprised 280 patients, organised into six age groups, ranging from 24 to 75+ years (median/mode age group 65–74 years, 52.5% male). Analysis of differences between Hba1c–complete and Hba1c–missing patients, on socio-demographic factors, revealed that the latter group were more likely to be older (72% aged >65 years), χ^2^ (1, *N* = 280) = 9.56, *p* < 0.01. HbA1c–missing patients were less disposed to high total cholesterol (>5 mmol/L), χ^2^ (1, *N* = 280) = 16.61, *p* < 0.001, and more prone to low HDL cholesterol (HDL < 1), χ^2^ (1, *N* = 280) = 146.07, *p* < 0.001, and a history of heart disease, χ^2^ (1, *N* = 280) = 8.92, *p* < 0.01. Sociodemographic and clinical parameters are shown in Table 1.

**Table 1.** Descriptive parameters on self-rated health.

	Total	Very good	Good	Fair	Bad
	Mean (SD)	Mean (SD)	Mean (SD)	Mean (SD)	Mean (SD)
Complete sample	(*N =* 159)	(*N =* 159)	(*N =* 159)	(*N =* 159)	(*N =* 159)
Multimorbidity (0–5 LTCs)	2.01 (1.36)	**1.00** ^a^ **(1.29)**	**1.54** ^b^ **(1.09)**	2.12 (1.19)	**3.12** ^a,b^ **(1.21)**
HbA1c (%)	7.31 (1.44)	7.10 (0.89)	7.50 (1.52)	7.35 (1.42)	6.87 (1.33)
Body mass index (BMI)	32.61 (6.42)	29.65 (4.61)	31.16 (5.10)	32.56 (6.46)	33.33 (5.98)
Total cholesterol (mmol/L)	4.18 (0.93)	4.17 (1.15)	4.23 (0.93)	4.04 (0.76)	4.08 (1.14)
HDL cholesterol (mmol/L)	1.24 (0.38)	1.26 (0.41)	1.33 (0.41)	1.17 (0.38)	1.13 (0.27)
Diastolic BP (mm Hg)	70.85 (11.06)	73.23 (6.46)	72.16 (9.84)	70.09 (10.89)	71.11 (13.10)
Systolic BP (mm Hg)	130.07 (16.94)	134.76 (14.22)	130.68 (15.32)	128.91 (15.52)	128.92 (19.12)
Males	(*N =* 84)	(*N =* 84)	(*N =* 84)	(*N =* 84)	(*N =* 84)
Multimorbidity (0–5 LTCs)	1.86 (1.36)	1.29 (1.38)	**1.35** ^c^ **(0.97)**	1.96 (0.98)	**3.00** ^c^ **(1.15)**
HbA1c (%)	7.41 (1.35)	7.02 (0.93)	7.52 (1.37)	7.66 (1.30)	6.88 (1.29)
Body mass index (BMI)	32.29 (5.68)	30.44 (5.67)	32.47 (4.70)	31.69 (5.30)	31.53 (6.82)
Total cholesterol (mmol/L)	4.01 (0.90)	3.92 (1.10)	4.18 (0.95)	3.95 (0.79)	3.45 (0.69)
HDL cholesterol (mmol/L)	1.13 (0.29)	1.22 (0.17)	1.16 (0.29)	1.03 (0.27)	1.08 (0.23)
Diastolic BP (mm Hg)	70.94 (10.83)	73.78 (7.48)	73.55 (10.92)	69.29 (11.74)	68.00 (8.59)
Systolic BP (mm Hg)	132.25 (14.93)	141.78 (12.00)	132.39 (12.37)	129.59 (9.30)	134.50 (18.07)
Females	(*N =* 75)	(*N =* 75)	(*N =* 75)	(*N =* 75)	(*N =* 75)
Multimorbidity (0–5 LTCs)	2.19 (1.35)	**0.67** ^d^ **(1.21)**	1.75 (1.19)	2.29 (1.39)	**3.23** ^d^ **(1.30)**
HbA1c (%)	7.20 (1.52)	7.20 (0.92)	7.48 (1.70)	7.00 (1.49)	6.86 (1.41)
Body mass index (BMI)	32.95 (7.15)	28.74 (3.24)	29.64 (5.20)	33.54 (7.56)	35.13 (4.58)
Total cholesterol (mmol/L)	4.37 (0.93)	4.46 (1.25)	4.30 (0.91)	4.15 (0.73)	4.72 (1.16)
HDL cholesterol (mmol/L)	1.36 (0.43)	1.30 (0.61)	1.53 (0.44)	1.32 (0.43)	1.18 (0.31)
Diastolic BP (mm Hg)	70.75 (11.36)	72.58 (5.66)	70.56 (8.31)	71.00 (10.03)	74.23 (16.21)
Systolic BP (mm Hg)	127.66 (18.68)	126.58 (12.79)	128.70 (18.15)	128.14 (20.60)	123.42 (19.17)

Samples sizes represent valid *N* (listwise). Mean values in bold reflect significant groups differences (*p* ⩽ 0.001): pairs of values with the same subscript differ significantly, based on MANOVA (post-hoc tests) (*p* ⩽ 0.001).

**Figure 2.**
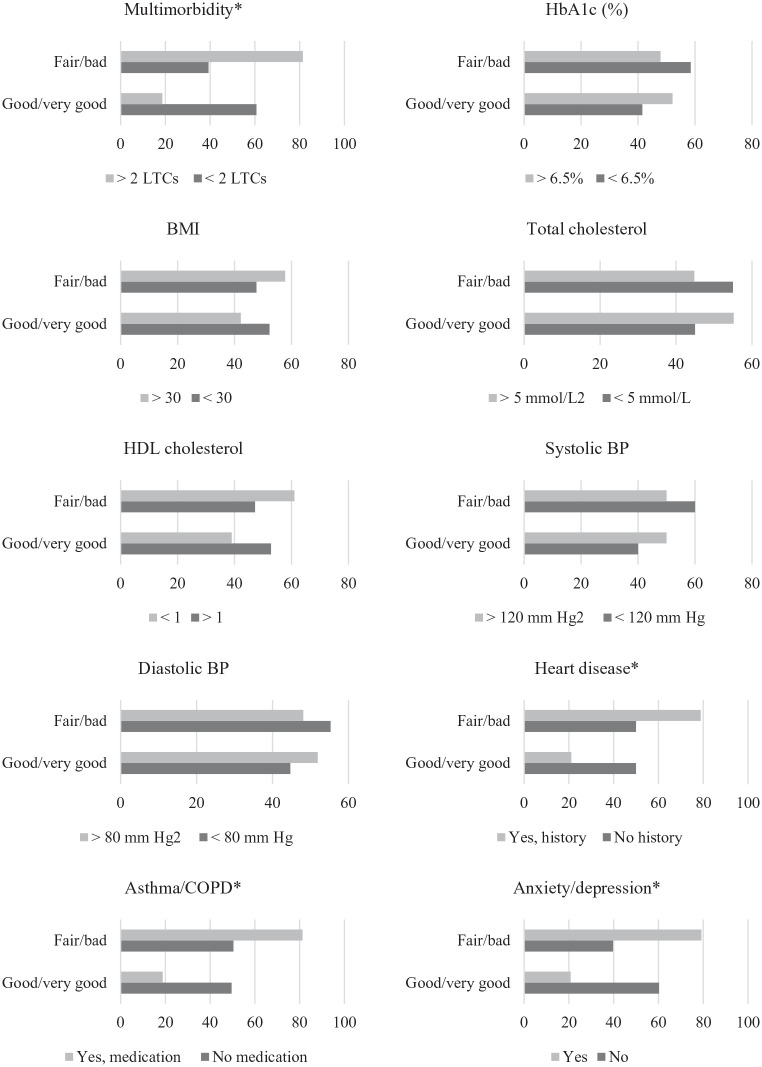
Frequencies for self-rated health by multimorbidity, HbA1c, BMI, cholesterol and individual comorbidities. Variables are dichotomised, to maximise cell frequencies. *p ⩽ 0.001.

There was a significant association between self-rated health and multimorbidity (Figure 2); patients with multimorbidity (⩾2 LTCs) were more likely to view their health as ‘fair/bad/very bad’ compared to those without multimorbidity, (χ^2^(1) = 45.22, *p* < 0.001). Patients with a history of heart disease were also more likely to report poor perceived health (χ^2^(1) = 11.07, *p* ⩽ 0.001). Negative views of health were also significantly more likely in patients with depression/anxiety (χ^2^(1) = 40.63, *p* < 0.001), and those on asthma/COPD medication (χ^2^(1) = 10.85, *p* ⩽ 0.001). Across the whole sample, 34.6% had been diagnosed with ⩾2 chronic conditions, 43.2% had Hba1c levels >6.5% (indicating poor glycaemic control) (Sherwani et al., 2016) and 54% perceived their health as ‘fair/bad/very bad’. Furthermore, 11.4% were taking asthma/COPD medication, 36.1% suffered from anxiety/depression, 13.6% had a history of heart disease and 61.8% were obese (BMI > 30). Just over 60% of patients had systolic readings >120 mm Hg, while 18.6% had diastolic values >80 mm: 65.4% had doctor-diagnosed hypertension. Almost half of the sample (48.6%) had a deficient HDL cholesterol level (<1), while 10.4% had high total cholesterol (>5 mmol/L). The mean Hba1c level (185 complete cases only), was 7.32% ± 1.44, which is over the recommended 6.5% cut-off for diagnosing diabetes. Patients reported an average of 2.01 ± 1.36 chronic conditions diagnosed. The mean BMI was 32.61 ± 6.42, denoting a sample that is generally overweight.

### Hypothesis 1: Logistic regression analysis

To reduce the risk of type 1 errors (false positives) a Bonferroni correction was applied. The resulting adjusted alpha level was circa *p* < 0.003, for the whole sample, and also each gender group. For consistency *p* < 0.001 was used as the default alpha level. Emerging logistic regression estimates are shown in Tables 2 to 4. Overall model parameters are displayed in Table 5. Demographic variables did not predict self-rated health (Model 1). Addition of multimorbidity variables (Model 2) resulted in good fit. The odds of reporting ‘fair/bad/very bad’ self-rated health was over 10 times higher in patients with three conditions (OR 10.53 (3.93–28.21)), and over 14 times greater in those with four illnesses (OR 14.87 (4.70–47.08)). The wide confidence intervals highlight reduced certainty, and the need for further verification with a larger sample. Addition of physiological risk factors, notably total/HDL cholesterol, HbA1c and systolic/diastolic blood pressure (Model 3), did not attenuate associations between self-rated health and multimorbidity; having three (OR 12.39 (4.45–34.48) and four (OR 15.72 (4.82–51.21) conditions predicted poor perceived health. Inclusion of individual comorbidities, specifically obesity, heart disease, anxiety/depression and asthma/COPD (Model 4), also failed to significantly alter associations between self-rated health and multimorbidity. The odds of reporting ‘fair/bad/very bad’ self-rated health remained over 10 times higher in patients with three conditions (OR 10.11 (3.36–30.40)). However, the odds were now just 10 times greater in patients with four illnesses (OR 10.58 (2.92–38.25)), reduced from over fifteen (Model 3). One comorbidity was significant; the odds of ‘fair/bad’ self-rated health was nearly five times greater in patients with anxiety/depression (OR 4.75 (2.38–9.49)).

**Table 2.** Hierarchical regression models predicting self-rated health from multimorbidity and covariates (complete sample).

*N* = 279	*B*	SE	*p*	OR	CI
Model 1					
Age	0.18	0.25	0.453	1.20	0.73–1.97
Social	−0.70	0.25	0.005	0.49	0.30–0.81
Gender	0.20	0.24	0.402	1.22	0.76–1.98
Model 2					
Age	0.09	0.28	0.734	1.10	0.63–1.91
Social	−0.84	0.28	0.003	0.43	0.24–0.75
Gender	−0.07	0.28	0.797	0.93	0.53–1.61
1 LTC	0.20	0.44	0.644	1.23	0.51–2.95
2 LTC	1.08	0.43	0.013	2.96	1.26–6.94
3 LTC	**2.35**	**0.50**	<0.001*	**10.53**	**3.93–28.21**
4 LTC	**2.70**	**0.58**	<0.001*	**14.87**	**4.70–47.08**
5 LTC	3.15	1.11	0.005	23.51	2.63–209.74
Model 3					
Age	0.02	0.30	0.930	1.02	0.56–1.85
Social	**−0.89**	**0.29**	**0.002**	**0.40**	**0.23–0.72**
Gender	−0.19	0.29	0.505	0.82	0.45–1.46
1 LTC	0.19	0.45	0.665	1.21	0.49–2.98
2 LTC	1.23	0.45	0.006	3.44	1.42–8.34
3 LTC	**2.51**	**0.52**	<0.001*	**12.39**	**4.45–34.48**
4 LTC	**2.75**	**0.60**	<0.001*	**15.72**	**4.82–51.21**
5 LTC	2.88	1.12	0.010	17.93	1.96–163.60
Total cholesterol >5 mmol/L	0.02	0.48	0.967	1.02	0.39–2.61
HDL cholesterol <1	0.07	0.37	0.846	1.07	0.51–2.23
HbA1c >6%	−0.71	0.38	0.064	0.49	0.23–1.04
Diastolic BP >80 mm Hg	−0.15	0.38	0.690	0.85	0.40–1.83
Systolic BP >120 mm Hg	−0.36	0.32	0.291	0.69	0.37–1.30
Model 4					
Age	0.09	0.33	0.774	1.10	0.56–2.13
Social	−0.62	0.32	0.054	0.53	0.28–1.01
Gender	−0.35	0.33	0.284	0.70	0.36–1.34
1 LTC	0.12	0.49	0.804	1.13	0.42–2.99
2 LTC	1.28	0.49	0.009	3.61	1.38–9.48
3 LTC	**2.31**	**0.56**	<0.001*	**10.11**	**3.36–30.40**
4 LTC	**2.35**	**0.65**	<0.001*	**10.58**	**2.92–38.25**
5 LTC	2.10	1.17	0.074	8.23	0.81–83.10
Total cholesterol >5 mmol/L	0.13	0.51	0.797	1.14	0.41–3.10
HDL cholesterol < 1	0.04	0.40	0.908	1.04	0.47–2.30
HbA1c >6%	−0.71	0.41	0.081	0.48	0.21–1.09
Diastolic BP >80 mm Hg	−0.41	0.42	0.331	0.66	0.28–1.52
Systolic BP >120 mm Hg	−0.23	0.34	0.493	0.78	0.39–1.55
Obese (BMI > 30), yes	0.16	0.32	0.622	1.17	0.62–2.22
Heart disease, yes	0.92	0.52	0.078	2.51	0.90–7.01
Depression/anxiety, yes	**1.56**	**0.35**	<0.001*	**4.75**	**2.38–9.49**
Asthma/COPD medication, yes	0.81	0.56	0.150	2.25	0.74–6.85

Boldfaced values denote significant regression coefficients (Bonferroni-adjusted, *p* < 0.001). *denotes significant based on bootstrapping, with 1000 resamples (Bonferroni-adjusted, *p* ⩽ 0.001).

**Table 3.** Hierarchical regression models predicting self-rated health from multimorbidity and covariates (males only).

*n* = 146	*B*	SE	*p*	OR	CI
Model 1					
Age	0.55	0.35	0.111	1.74	0.88–3.47
Social	−0.54	0.34	0.117	0.58	0.29–1.14
Model 2					
Age	0.82	0.40	0.041	2.27	1.03–5.00
Social	−1.06	0.40	0.008	0.34	0.15–0.75
1 LTC	−0.24	0.50	0.634	0.78	0.29–2.11
2 LTC	0.48	0.53	0.369	1.61	0.56–4.61
3 LTC	**2.17**	**0.68**	⩽0.001*	**8.81**	**2.30–33.64**
4 LTC	3.21	1.13	0.005	25.00	2.68–232.88
Model 3					
Age	0.68	0.46	0.154	1.95	0.77–4.88
Social	−1.12	0.43	0.010	0.32	0.13–0.76
1 LTC	−0.21	0.52	0.681	0.80	0.28–2.25
2 LTC	0.67	0.56	0.239	1.95	0.64–5.96
3 LTC	**2.54**	**0.72**	<0.001*	**12.78**	**3.06–53.26**
4 LTC	3.24	1.17	0.006	25.65	2.58–225.15
Total cholesterol >5 mmol/L	0.21	0.72	0.764	1.24	0.30–5.15
HDL cholesterol <1	0.71	0.50	0.157	2.03	0.75–5.47
HbA1c >6%	−0.44	0.53	0.408	0.64	0.22–1.83
Diastolic BP >80 mm Hg	−0.44	0.57	0.437	0.63	0.20–1.97
Systolic BP >120 mm Hg	−0.46	0.45	0.309	0.62	0.25–1.53
Model 4					
Age	0.68	0.53	0.201	1.98	0.69–5.68
Social	−1.08	0.47	0.023	0.33	0.13–0.86
1 LTC	−0.27	0.60	0.648	0.75	0.23–2.49
2 LTC	0.89	0.63	0.160	2.43	0.70–8.42
3 LTC	**2.52**	**0.79**	⩽0.001*	**12.46**	**2.64–58.84**
4 LTC	2.64	1.24	0.034	14.01	1.22–160.20
Total cholesterol >5 mmol/L	0.95	0.82	0.247	2.60	0.51–13.13
HDL cholesterol <1	0.71	0.54	0.186	2.05	0.70–5.95
HbA1c >6%	−0.87	0.59	0.143	0.41	0.13–1.34
Diastolic BP >80 mm Hg	−0.92	0.68	0.178	0.39	0.10–1.52
Systolic BP >120 mm Hg	−0.59	0.51	0.249	0.55	0.20–1.51
Obese (BMI >30), yes	−0.41	0.48	0.391	0.66	0.25–1.70
Heart disease, yes	0.29	0.65	0.653	1.34	0.37–4.81
Depression/anxiety, yes	**2.05**	**0.58**	<0.001*	**7.83**	**2.51–24.42**
Asthma/COPD medication, yes	0.85	0.79	0.281	2.36	0.49–11.26

Boldfaced values denote significant regression coefficients (Bonferroni-adjusted, *p* ⩽ 0.001). *denotes significant based on bootstrapping, with 1000 resamples (Bonferroni-adjusted, *p* ⩽ 0.001). The ‘5 LTC’ dummy variable is excluded due to low cell frequencies.

**Table 4.** Hierarchical regression models predicting self-rated health from multimorbidity and covariates (females only).

*n* = 133	*B*	SE	*p*	OR	CI
Model 1					
Age	−0.21	0.36	0.561	0.80	0.39–1.65
Social	−0.88	0.37	0.017	0.41	0.19–0.85
Model 2					
Age	−0.56	0.41	0.172	0.56	0.25–1.27
Social	−0.83	0.40	0.040	0.43	0.19–0.96
1 LTC	−0.54	0.66	0.413	0.58	0.15–2.12
2 LTC	0.64	0.56	0.249	1.91	0.63–5.77
3 LTC	1.78	0.66	0.007	5.96	1.61–22.03
4 LTC	1.87	0.70	0.008	6.53	1.63–26.11
Model 3					
Age	−0.52	0.42	0.222	0.59	0.25–1.37
Social	−0.92	0.42	0.030	0.39	0.17–0.91
1 LTC	−0.68	0.70	0.328	0.50	0.12–1.99
2 LTC	0.76	0.60	0.210	2.14	0.65–7.05
3 LTC	1.82	0.70	0.009	6.18	1.56–24.40
4 LTC	1.93	0.72	0.008	6.92	1.66–28.80
Total cholesterol >5 mmol/L	0.00	0.64	0.996	1.00	0.28–3.52
HDL cholesterol <1	−0.24	0.57	0.671	0.78	0.25–2.42
HbA1c >6%	−0.89	0.57	0.119	0.40	0.13–1.26
Diastolic BP >80 mm Hg	0.43	0.58	0.454	1.54	0.49–4.85
Systolic BP >120 mm Hg	−0.14	0.44	0.747	0.86	0.36–2.07
Model 4					
Age	−0.40	0.49	0.414	0.55	0.25–1.75
Social	−0.38	0.53	0.470	0.68	0.24–1.92
1 LTC	−0.91	0.84	0.284	0.40	0.07–2.12
2 LTC	0.79	0.73	0.279	2.21	0.52–9.33
3 LTC	1.84	0.80	0.022	6.34	1.31–30.66
4 LTC	1.49	0.86	0.083	4.46	0.82–24.27
Total cholesterol >5 mmol/L	0.03	0.70	0.962	1.03	0.26–4.12
HDL cholesterol <1	−0.74	0.65	0.257	0.47	0.13–1.71
HbA1c >6%	−0.89	0.64	0.165	0.41	0.11–1.44
Diastolic BP >80 mm Hg	0.17	0.66	0.799	1.18	0.32–4.38
Systolic BP >120 mm Hg	0.13	0.51	0.795	1.14	0.41–3.14
Obese (BMI >30), yes	0.78	0.49	0.110	2.19	0.83–5.75
Heart disease, yes	2.21	1.18	0.061	9.14	0.90–92.65
Depression/anxiety, yes	1.48	0.51	0.004	4.39	1.59–12.12
Asthma/COPD medication, yes	2.14	1.15	0.064	8.52	0.87–82.64

There are no significant regression coefficients (all *p*’s > 0.001, Bonferroni-adjusted). There are also no significant coefficients based on bootstrapping, with 1000 resamples (all *p*’s > 0.001, Bonferroni-adjusted). The ‘5 LTC’ dummy variable is excluded due to low cell frequencies.

**Table 5.** Overall model parameters.

Model indices	Model 1			Model 2			Model 3			Model 4		
	χ^2^	df	*p*	χ^2^	df	*p*	χ^2^	df	*p*	χ^2^	df	*p*
Complete sample												
Step	8.849	3	0.031	**59.767**	**5**	**<0.001**	9.208	5	0.101	**30.875**	**4**	**<0.001**
Block	8.849	3	0.031	**59.767**	**5**	**<0.001**	9.208	5	0.101	**30.875**	**4**	**<0.001**
Model	8.849	3	0.031	**68.616**	**8**	**<0.001**	**77.824**	**13**	**<0.001**	**108.698**	**17**	**<0.001**
Hosmer and Lemeshow test	7.260	6	0.297	2.834	7	0.900	7.412	8	0.493	10.049	8	0.262
Males												
Step	4.608	2	0.100	**30.155**	**4**	**<0.001**	9.787	5	0.082	**19.223**	**4**	**⩽0.001**
Block	4.608	2	0.100	**30.155**	**4**	**<0.001**	9.787	5	0.082	**19.223**	**4**	**⩽0.001**
Model	4.608	2	0.100	**34.763**	**6**	**<0.001**	**44.550**	**11**	**<0.001**	**63.773**	**15**	**<0.001**
Hosmer and Lemeshow test	0.268	2	0.874	1.563	7	0.980	5.243	7	0.630	6.052	8	0.641
Females												
Step	6.589	2	0.037	**21.352**	**4**	**<0.001**	4.360	5	0.499	**27.855**	**4**	**<0.001**
Block	6.589	2	0.037	**21.352**	**4**	**<0.001**	4.360	5	0.499	**27.855**	**4**	**<0.001**
Model	6.589	2	0.037	**27.942**	**6**	**<0.001**	**32.301**	**11**	**⩽0.001**	**60.157**	**15**	**<0.001**
Hosmer and Lemeshow test	4.172	2	0.124	5.709	8	0.680	9.833	8	0.277	5.228	8	0.733

Model 1 (demographics), Model 2 (multimorbidity added), Model 3 (HbA1c, cholesterol and blood pressure added), Model 4 (comorbidities added). Model 4 includes all predictor variables. Boldfaced values indicate significance. Hosmer and Lemeshow goodness-of-fit test assesses whether observed frequencies reflect expected frequencies in subgroups (with different predicted probabilities) within the model population. *p*-values < 0.05 indicate poor fit, albeit *p* > 0.05 does not necessarily indicate good fit.

The regression was repeated separately by gender. In males, inclusion of multimorbidity variables (Model 2) produced a significant model. The odds of reporting ‘fair/bad/very bad’ self-rated health was about eight times higher in patients with three conditions (OR 8.81 (2.30–33.64)). Adding physiological risk factors (cholesterol, HbA1c, blood pressure) (Model 3) did not significantly affect relations between perceived health and multimorbidity, albeit the odds of ‘fair/bad/very bad’ self-rated health increased slightly in patients with three diseases (OR 12.78 (3.06–53.26)). Including individual comorbidities (Model 4), also failed to attenuate the association between perceived health and multimorbidity (OR 12.46 (2.64–58.84)). The odds of ‘fair/bad’ self-rated health was nearly eight times higher in patients with anxiety/depression (OR 7.83 (2.51–24.42)). As in the overall sample, wide confidence intervals with males indicated reduced certainty. In females, adding multimorbidity (Model 2) did not produce any significant associations with perceived health (all *p*’s > 0.001). These null results persisted after including physiological risk factors (Model 3) and comorbidities (Model 4). Having three or four conditions approached significance in Models 2 and 3 (all *p*’s < 0.009, but >0.001).

### Sensitivity analysis

The data was reanalysed with and without expectation maximisation applied to missing HbA1c data. Results generally supported the primary analysis. The odds of reporting ‘fair/bad/very bad’ self-rated health remained significantly higher in patients with three (OR 13.21 (3.14–55.54)) or four conditions (OR 24.72 (3.90–156.47)). As before, the odds of ‘fair/bad’ self-rated health was significantly higher in patients with anxiety/depression (OR 4.27 (1.80–10.15)). Results by gender also echoed the primary analysis. In males, the odds of reporting ‘fair/bad/very bad’ self-rated health was higher in patients with three conditions (OR 22.65 (2.78–184.08), albeit not at the Bonferroni-adjusted alpha level of *p* < 0.003 (observed *p* = 0.004). A similar pattern emerged for depression/anxiety. In females multimorbidity did not produce any significant associations with perceived health (all *p*’s > 0.001). Overall, *sensitivity analysis* suggests that the primary analysis was robust.

### Hypothesis 2: Structural equation modelling

The following thresholds were used to assess model fit (Hooper et al., 2008): model chi-square χ^2^ (CMIN) (*p* > 0.05), χ^2^ (CMIN)/df < 2.00, root mean square error of approximation (RMSEA) < 0.07, comparative fit index (CFI) ⩾ 0.95, Tucker and Lewis Index (TLI) ⩾ 0.95 and normed fit index (NFI) ⩾ 0.95. Due to ambiguity regarding interpretation of parsimony fit indices, specifically the parsimony normed fit index (PNFI), these criteria were not used (Hooper et al., 2008). Furthermore, IBM AMOS SPSS (Version 26) does not generate root mean square residual (RMR) or standardised root mean square residual (SRMR) values. Finally, observed TLI values were subject to less stringent interpretation because this index can be lower than expected (i.e. <0.95) when small samples are employed, indicating poor fit, while other criteria denote good fit (Tabachnick and Fidell, 2007). An initial model was tested, consistent with Hypothesis 1: multimorbidity was allowed to affect HbA1c, both directly and indirectly, mediated by self-rated health; the direct and indirect effects of multimorbidity on BMI were also tested, again with perceived health treated as a mediator.

Goodness-of-fit parameters for this initial model were; χ^2^ (CMIN) = 4.34, df = 1, *p* = 0.037, χ^2^ (CMIN)/df = 4.34, RMSEA = 0.11, CFI = 0.96, TLI = 0.76 and NFI = 0.95. Despite the satisfactory CFI and NFI values, the CMIN (*p* < 0.05), χ^2^ (CMIN)/df (>2) and RMSEA (>0.07), all indicated poor fit. The Bollen-Stine bootstrap result, based on 200 resamples, also indicated a poor fitting model (*p* = 0.035). Thus, it was decided to perform post-hoc modifications. The data was reanalysed using the AMOS specification-search function, to test out different models. Specification-search provides a mechanism for systematically evaluating the fit of multiple candidate models, in order to identify the best fitting framework.

**Figure 3.**
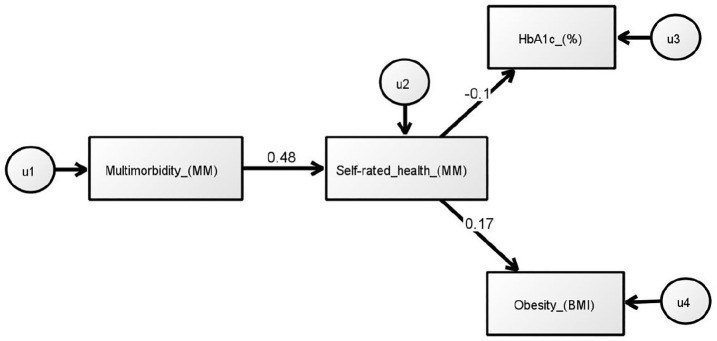
Optimal structural equation model, using search specification (AMOS, v.26). The SRH → HbA1c pathway is not significant (*p* > 0.05).

Of the 10 models generated one was chosen based primarily on the χ^2^ (CMIN)/df value. This model is presented in Figure 3. Maximum likelihood estimations are shown in Table 6. Multiple fit criteria indicated good fit: χ^2^ (CMIN) = 5.10, df = 3, *p* = 0.164, χ^2^ (CMIN)/df = 1.70, RMSEA = 0.05, CFI = 0.97, TLI = 0.95 and NFI = 0.94 (requirements; CMIN (*p* > 0.05), χ^2^ (CMIN)/df (<2), RMSEA (<0.07), CFI (⩾0.95) and TLI (⩾0.95)). Although the NFI was borderline (<0.95), this criterion is sensitive to sample size; it tends to underestimate fit for samples <200 (Hooper et al., 2008). The Bollen-Stine bootstrapping test was non-significant, denoting good fit (*p* = 0.159): the model provided a better fit in 169 bootstrap samples, fit equally well in zero samples and gave a worse fit or failed to fit in just 31 samples.

**Table 6.** Maximum likelihood estimation results.

			Estimate	SE	CR	*p*	Label
Self-rated health	←	Multimorbidity	0.485	0.033	9.254	***	Supported
HbA1c (glycaeted haemoglobin)	←	Multimorbidity	0.000	−	−	−	Not significant
Body mass index	←	Multimorbidity	0.000	−	−	−	Not significant
HbA1c (glycaeted haemoglobin)	←	Self-rated health	−0.095	0.081	−1.598	0.110	Not significant
Body mass index	←	Self-rated health	0.176	0.436	2.980	0.003	Supported

Estimates are standardised regression weights (default model). ***indicate *p* < 0.001. HbA1c is calibrated in percentages, not mmol/mol.

It was hypothesised that multimorbidity is *indirectly* related to both HbA1c and BMI, mediated by self-rated health. The model showed that multimorbidity was associated with perceived health; patients with multiple conditions reported poorer health evaluations (β = 0.48, *p* < 0.001). The direct associations of multimorbidity with HbA1c and BMI were not statistically significant. Self-rated health was related to body weight; patients with more negative assessments had higher BMI scores (β = 0.17, *p* = 0.003). However, perceived health was unrelated to HbA1c, negating the proposition that the former mediates relations between multimorbidity and glycaemic control. Squared multiple correlations showed the default model explained 23.5% and 3.1% of the variance in self-rated health and BMI, respectively. Confidence in the fit of the model was enhanced by the CFI value of 0.97 (⩾0.95), as this fit index is least affected by sample size (Hooper et al., 2008). The TLI value (⩾95) also indicated good fit, despite a propensity to denote poor fit when modest sample sizes are used (Tabachnick and Fidell, 2007).

## Discussion

This is the first study to assess how self-rated health relates to multimorbidity, glycaemia and body weight, in adult patients with type 2 diabetes. Previous studies on self-rated health and multimorbidity have focused on generic or healthy populations (Bustos-Vazquez et al., 2017; Ishizaki et al., 2019; Mavaddat et al., 2014; Song et al., 2018). Research specifically examining people with diabetes is rare. The present study tested two propositions: that greater multimorbidity is associated with poorer self-rated health (Hypothesis 1) and multimorbidity is indirectly related to both glycaemia and BMI, mediated by self-rated health (Hypothesis 2). Data analysis revealed support for Hypothesis 1 and partial verification of Hypothesis 2.

### Hypothesis 1

Self-rated health was associated with multi-morbidity, after adjusting for HbA1c, obesity, other physiological risk factors and individual comorbidities. Patients with multimorbidity (three or four conditions) were multiple times more likely to report poor perceived health, echoing previous research (Bustos-Vazquez et al., 2017; Ishizaki et al., 2019; Song et al., 2018). Gender played an important role; while multimorbidity predicted self-rated health in males, the data for females was ambiguous. This asymmetry reflects previous research showing a more pronounced link between multimorbidity and perceived health in men compared to women (Mavaddat et al., 2014). However, some research has found no gender differences (Galenkamp et al., 2011). One possible reason for these inconsistencies is that relations between multimorbidity and self-rated health are underpinned by complex human judgements about the seriousness of one or multiple illnesses, which may vary by gender, and also be specific to patients with a particular disease (Idler and Benyamini, 1997).

A review of men and women’s adjustment to diabetes-related challenges found that male patients live more effectively with diabetes, experiencing less depression and anxiety (Siddiqui et al., 2013). However, other research suggests depression is the main reason multimorbid men perceive poor health, whereas depression is just one of many factors influencing multimorbidity and self-rated health in females (Assari et al., 2019). Another possible explanation is that males experience more severe multimorbidity than females, leading to poorer health assessments (Idler and Benyamini, 1997). The present data suggests males reported fewer illnesses compared to females (see Table 1). Nevertheless, male patients may experience more severe symptoms due to less use of diabetes health services (Siddiqui et al., 2013). Additionally, diminished social activity has been associated with poor self-rated health in older men (Caetano et al., 2013). Less social activity in multimorbid men may precipitate more pessimistic assessments of general wellbeing. Overall, more research is needed to better understand the complex biopsychosocial factors underpinning gender differences in multimorbidity and self-rated health.

### Hypothesis 2

Although multimorbidity predicted self-rated health, neither was associated with HbA1c, contradicting previous research (Abualula et al., 2018; Nielsen et al., 2011). However, the present data supports a recent systematic review in which the majority of studies found no multimorbidity–HbA1c connection (Chiang et al., 2018). Self-rated health did mediate multimorbidity–BMI relations, whereby presence of multiple diseases and being overweight were underpinned by poor perceived health. Past research has established associations between body weight and poor self-rated health (Wang and Arah, 2015); the present findings suggest the role of perceived health goes further, in type 2 diabetes, partly explaining the obesity–multimorbidity relationship (Madlock-Brown and Reynolds, 2019).

Although there is abundant evidence on obesity-related multimorbidity (Agborsangaya et al., 2013; Agrawal and Agrawal, 2016; Booth et al., 2014; Kivimaki et al., 2017; Madlock-Brown and Reynolds, 2019), no study has examined self-rated health in this context, especially amongst people with diabetes. Previous research indicates obesity–related multimorbidity depends on socio-cultural characteristics (Madlock-Brown and Reynolds, 2019). The present findings suggest self-rated health is also an important underlying factor. The term ‘obesity-related multimorbidity’, used to describe common obesity-related groupings of multimorbidities in a recent paper (Madlock-Brown and Reynolds, 2019), is appropriate here since body weight was associated with multimorbidity, albeit indirectly, mediated by perceived health. Patients with multiple illnesses are perhaps more likely to view their health as deteriorating, and consequently lose motivation to adopt weight management behaviours (Idler and Benyamini, 1997).

### Limitations

Expectation-maximisation was used to estimate missing HbA1c values because this method is generally considered superior to other techniques for resolving missing data (e.g. mean substitution), which generate biased estimates. Nevertheless, how these estimations affected the findings is unclear. Sensitivity analyses did not produce any significant changes in the interpretation of the data from logistic regression analysis. Nevertheless, the marginally less robust alpha level observed in males (*p* = 0.004) may point to the need for future research to address the source of this discrepancy. Simulation studies comparing multiple approaches of resolving missing data may be necessary. Expectation maximisation converges to a local solution, necessitating further research to verify the present results using alternative methods for managing missing data. Another problem is failure to assess the severity, duration and type of multimorbidities. For example, two people with diabetes experiencing exactly the same number and type of chronic conditions may nevertheless develop highly divergent self-perceptions of health, due to variation in symptom severity.

Extending the current observations to other chronic disease populations is an important avenue for future research. In particular, future studies should examine the extent to which the findings generalise to patients with type 1 diabetes (who are typically younger). The HbA1c test is also used to evaluate glycaemic control and predict complications in type 1 patients (Sherwani et al., 2016). Future research should test mediation models in type 1 patients, to see how self-rated health affects relations between multimorbidity and HbA1c. Patients in this study were in better glycaemic condition than expected (most generating HbA1c levels <6.5%). They also reported less multimorbidity (just 34.6% had ⩾2 conditions, compared to 90% reported elsewhere) (Teljeur et al., 2013). Thus, the current sample isn’t representative. Furthermore, HbA1c levels depend on diabetes history, medication regimes and short/long-term insulin dosage (Sherwani et al., 2016). It is therefore essential for future research to control for these confounders.

### Implications

The current findings may have implications for patient care. In clinical settings, self-rated health may be measured as part of routine patient monitoring, and/or during initial consultation, or registration. Since detection of multimorbidity continues to present a challenge for physicians (diagnostic uncertainty) (Hausmann et al., 2019), self-rated health can be used as a reliable marker for multimorbidity in people with diabetes, ancillary to other diagnostic criteria used by doctors. Perceived health arguably captures the full array of illnesses a person is experiencing, including undiagnosed morbidity still at a preclinical or prodromal stage (Idler and Benyamini, 1997). Thus, a patient reporting poor self-rated health can be referred for further clinical testing, to identify any disease clusters. This scenario is primarily applicable to men, given the tenuous link between perceived health and multimorbidity in women. Self-rated health might also help identify patients with obesity-related multimorbidity (Madlock-Brown and Reynolds, 2019). In this context it is possible efforts to improve self-rated health in patients with multimorbidity could also support attempts at weight control (which could further improve outcomes for people with T2DM), but are unlikely to have a substantive impact on HbA1c.

## Conclusion

The relationship between multimorbidity and self-rated health is well established. This is the first investigation of how self-rated health relates to multimorbidity, glycaemia and body weight, specifically in adult patients with type 2 diabetes. The findings indicate patients with multimorbidity have poorer self-rated health. Furthermore, perceived health underpins obesity-related multimorbidity in this clinical population, albeit neither self-rated health nor multimorbidity predicts HbA1c. Thus, how patients with type 2 diabetes view their health is unconnected to glycaemic control. Overall, this study highlights the potential of self-rated health to explain relationships between multimorbidity and BMI, but not glycaemic control, in people with type 2 diabetes. The role of self-rated health in glycaemia seems more obfuscated than was previously thought, and entail underlying mechanisms that have yet to be fully understood.
